# Development of Safer Gene Delivery Systems to Minimize the Risk of Insertional Mutagenesis-Related Malignancies: A Critical Issue for the Field of Gene Therapy

**DOI:** 10.5402/2012/616310

**Published:** 2012-11-22

**Authors:** Gaetano Romano

**Affiliations:** Department of Biology, College of Science and Technology, Temple University, Bio-Life Science Building, Suite 456, 1900 N. 12th Street, Philadelphia, PA 19122, USA

## Abstract

Integrating gene delivery systems allow for a more stable transgene expression in mammalian cells than the episomal ones. However, the integration of the shuttle vector within the cellular chromosomal DNA is associated with the risk of insertional mutagenesis, which, in turn, may cause malignant cell transformation. The use of a retroviral-derived vector system was responsible for the development of leukemia in five children, who participated in various clinical trials for the treatment of severe combined immunodeficiency (SCID-X1) in France and in the United Kingdom. Unfortunately, the hematological malignancy claimed the life of one patient in 2004, who was enrolled in the French clinical trial. In addition, adeno-associated-viral-(AAV-) mediated gene transfer induced tumors in animal models, whereas the Sleeping Beauty (SB) DNA transposon system was associated with insertional mutagenesis events in cell culture systems. On these grounds, it is necessary to develop safer gene delivery systems for the genetic manipulation of mammalian cells. This paper discusses the latest achievements that have been reported in the field of vector design.

## 1. Introduction

 Gene transfer technology requires the introduction of recombinant genetic elements into human cells and holds a considerable therapeutic potential for the treatment of a wide variety of pathological conditions, such as cancer, genetic disorders, neurological illnesses, diabetes, infectious diseases, and cardiovascular maladies [1–7]. The gene-based treatments both of cancer and infectious diseases may require only a transient expression of the recombinant genetic elements, which have the function to destroy either neoplastic tissues, or cells that harbor an infectious agent [3, 4]. Conversely, genetic disorders, neurological illnesses and cardiovascular maladies need a long-term transgene expression, as the treatment of these maladies envisions the introduction of functional copies of certain genes in the attempt to correct the phenotype of the disease [3, 4]. A long-term transgene expression is also required for autologous T lymphocytes that are genetically engineered to express recombinant T cell receptors, which may impart binding specificity either for neoplastic markers or infected cells [5–7]. To this end, gene delivery systems that integrate their genome into the target cell chromosomal DNA allow for a more stable and long-lasting transgene expression [3, 4]. Various types of gene delivery systems are currently available [3, 4, 8–16]. The most common gene transfer models derive from retroviruses, lentiviruses, adenoviruses, adeno-associated viruses (AAV) and so-called non-viral vector systems [3, 4, 8–16]. Adenoviruses and most of nonviral-derived vectors are episomal gene transfer models [3, 4, 13, 14], whereas vectors based on retroviruses, lentiviruses, AAV, Sleeping Beauty (SB) DNA transposon system, and *Steptomyces* bacteriophage integrase ΦC31 have integrative properties [3, 4, 12, 14–16]. Sleeping Beauty (SB) DNA transposon system and *Streptomyces* bacteriophage integrase ΦC31 are non-viral-derived gene transfer systems that only require a plasmid DNA transfection for the delivery of their transgene, which is then stably integrated within the target cell genome [14]. 

 Retroviral-based vector systems were utilized in the first two phase I gene therapy clinical trials, which were conducted in the United States of America between 1989 and 1990 [3, 4, 8–10]. One clinical trial dealt with the treatment of adenosine deaminase (ADA) deficiency, which is a genetic disorder leading to immunodeficiency [3, 4, 9, 10]. A retroviral vector carrying the functional copy of adenosine deaminase was used for the *ex vivo* gene transduction of autologous bone marrow-derived hematopoietic stem cells, which were subsequently reinfused back into the two young patients who were enrolled in this trial [3, 4, 9, 10]. The other clinical study used a retroviral-encoded neomycin resistance gene to trace autologous human tumor-infiltrating T lymphocytes in five patients with advanced melanoma [3, 4, 8–10]. Both gene therapy clinical trials demonstrated to be feasible and safe in patients. Moreover, a long-term clinical benefit was reported in the two young patients who participated in the first human gene therapy clinical trial for the treatment of ADA deficiency. The functions of the immune system were restored in both patients [3, 4, 9, 10]. Remarkably, the expression of the recombinant ADA gene was observed in 20% of lymphocytes of one patient ten years after the last infusion of transduced bone-marrow-derived hematopoietic stem cells [10]. Naturally, this initial success prompted for the worldwide submission of hundreds of phase I and phase II human gene therapy protocols, which utilized various viral and nonviral gene delivery models for the treatment of monogenic disorders and cancer [3, 4]. However, the majority of these clinical trials provided disappointing results, as the design of the various gene transfer systems were not sufficiently adequate to support efficacious human gene therapy protocols in the clinical setting [3, 4]. Common problems were related to transgene silencing following the genetic manipulation of target cells and/or mediocre transduction efficiency [3, 4]. In addition, host immune responses to the vector systems and/or transduced cell populations constituted a very critical issue both in terms of safety and efficacy for the gene-based interventions [3, 4, 11–15, 17]. A young patient affected by a partial ornithine transcarbamylase deficiency died because of an acute inflammatory reaction, which was caused by a massive intrahepatic infusion of adenoviral vector particles carrying the functional copy of the defective gene [3, 13, 18]. This gene-based clinical trial was conducted in the United States of America in September 1999 and represented the first severe setback for gene therapy programs, which underwent austere public scrutiny [3, 13, 18]. Therefore, the field of vector design had to tackle a variety of critical issues to control host humoral and/or cytotoxic T cell (CTL) immune responses against viral vector particles and/or transduced cell populations [3, 4, 11–15, 17]. Furthermore, it was necessary to improve the production of viral and non-viral vector stocks, enhance the efficiency of *ex vivo* and *in vivo* gene transduction of several cell types, optimize transgene expression levels after gene transduction, and stabilize the duration of transgene expression in target cells [3, 4, 11–15, 17]. On these grounds, viral promoters were genetically modified in order to minimize *de novo* methylation of CpG-rich islands, which is one of the factors that are responsible for transgene silencing [3, 4, 11–15, 17]. Stronger enhancers were utilized to sustain higher levels of transgene expression in transduced cell populations [3, 4, 11–15, 17]. Lastly, protocols for the *in vitro* gene transduction of human hematopoietic stem cells were optimized [11–17]. These strategies contributed to the development of more efficient vector systems for gene transfer modalities both in human gene therapy protocols and preclinical studies [11–17]. Optimized gene transfer protocols were utilized in the field of stem cell research for the genetic manipulation of human and mouse embryonic stem (ES) cells, several types of adult stem cells, and, more recently, the production of induced pluripotent stem (iPS) cells, which may derive from every type of either human or animal somatic cell [19–33]. Indeed, gene transfer technology has emerged as a very useful tool to support stem cell research over the last decade [18–32]. The merging of gene transfer technology and stem cell research may have important implications in the field of regenerative medicine, which is aiming at developing novel therapeutic approaches for the treatment of neurodegenerative diseases, diabetes, and cardiovascular disorders [19–33]. So far, the combination between gene therapy and stem cell research was applied for the treatment of hematological maladies that derive from genetic disorders, such as ADA deficiency and severe combined immunodeficiency (SCID)-X1 [1–4]. 

On one hand, the integration of the shuttle vector within the chromosomal DNA of the target cell is one of the requirements for a long-term transgene expression. On the other hand, however, integrative gene transfer models have a potentially dangerous downside, as they are associated with the risk of insertional mutagenesis, which may eventually result in the development of malignancies [3, 4, 11, 12, 14, 15, 17]. The integration of the shuttle vector may tamper with the natural chromosomal arrangement of the target cell and trigger a variety of events leading to the establishment of a transformed cell phenotype [3, 4]. Unfortunately, retroviral-mediated gene transfer was responsible for the development of leukemia in five young subjects who participated in clinical trials for the treatment of SCID-X1 in France and in the United Kingdom [1, 2, 34–44]. The various outcomes reported in the SCID-X1 clinical trials and the mechanisms leading to the development of insertional mutagenesis-induced carcinogenesis will be discussed in greater detail in the next section. This paper will also describe the current strategies adopted to minimize and possibly prevent insertional mutagenesis-related events in patients. 

## 2. Mechanisms of Insertional Mutagenesis-Induced Carcinogenesis in the SCID-X1 Clinical Trials

SCID-X1 is an inherited genetic disorder that leads to immunodeficiency and is associated with the loss of the common *γ* chain (*γ*c) cytokine receptor subunit [36, 45, 46]. The absence of the *γ*c subunit impairs the biological and biochemical functions of the cellular receptors that bind a variety of interleukins, such as IL-2, IL-4, IL-7, IL-9, IL-15, and IL-21 [45, 46]. The impaired cellular receptors fail to interact with a variety of interleukins, which are important regulators of cell proliferation, growth, survival, and differentiation [42, 43]. For this reason, early lymphoid progenitors become unresponsive to the previously mentioned interleukins and, for this reason, they are not able to differentiate into B, T, and natural killer (NK) cells [45, 46]. The deprivation of these three cell types in the hematopoietic compartment results in a severe host immunodeficiency [45, 46]. 

A retroviral-encoded *γ*c subunit was used in the various gene therapy clinical trials for the treatment of SCID-X1, which were initially conducted in France [37] and then in the United Kingdom [42, 47], Australia [48], and United States of America [49]. Beneficial clinical outcomes were reported in all of these gene-based trials [42, 47–50]. Autologous CD34^+^ bone marrow-derived cells were extracted from the patients and transduced *in vitro* with a retroviral-based vector system. The retroviral vector transduction was very efficient and the expression of the recombinant *γ*c subunit rendered autologous CD34^+^ bone-marrow cells susceptible to the influence of IL-2, IL-4, IL-7, IL-9, IL-15, and IL-21 [45]. The genetically modified autologous bone-marrow cells were reinfused into the patients, in the attempt to correct the phenotype of the disease. The French clinical trial was conducted in 2000 and initially reported a considerable success, as the immune system functions were restored in 9 out of 10 patients [45]. However, four of these patients developed a leukemia-like illness in the following months [1–3, 34–44]. The hematological malignancy was first observed in two patients after periods of 30 and 34 months [1–3, 37, 43], whereas the other two patients came down with leukemia in subsequent months [2, 34, 36, 43]. Sadly, one of the first two patients who developed leukemia had a relapse of the disease and died in October 2004 [2, 38, 39]. An additional fifth case of leukemia was reported in a child who participated in the British SCID-X1 trial that was conducted in 2005 [42]. The British and the Australian gene therapy clinical trials utilized lower amounts of transduced autologous CD34^+^ hematopoietic stem cells for infusion into the patients, with the intent to reduce the risk of insertional mutagenesis-induced malignancies [42, 47, 48]. Unfortunately, this strategy was not particularly effective, as shown by the fifth case of leukemia reported in the British SCID-X1 clinical trial [42]. 

**Figure 1:**
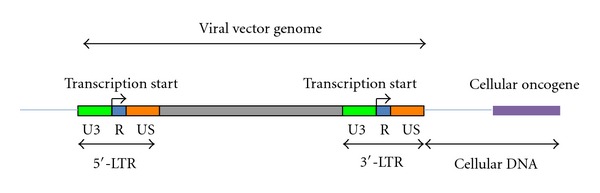
This figure shows the first mechanism for cellular oncogene expression, which takes place through transcriptional activation promoter that is mediated by the retroviral 3′-long terminal repeat (LTR). The bent arrows depict the transcription start site of the 5′- and 3′-LTR.

**Figure 2:**
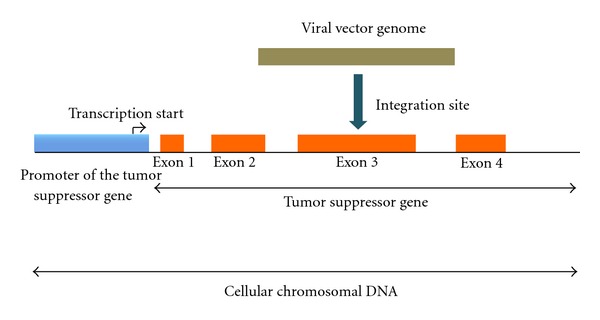
This figure displays the mechanism of tumor suppressor gene silencing, which can be mediated by the retroviral vector insertion within an exon of the tumor suppressor gene.

**Figure 3:**
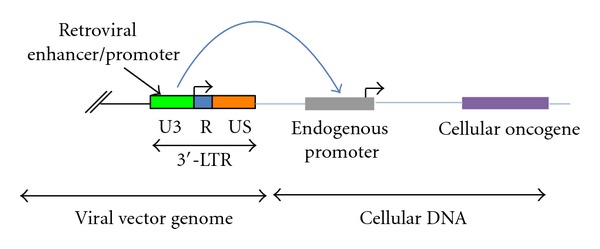
This figure reports the second mechanism for cellular oncogene expression, which is caused by the long-range interaction of the retroviral enhancer and the endogenous promoter of the cellular oncogene. The blue block arrow represents the interaction between the retroviral enhancer and the endogenous promoter of the cellular oncogene.

There are two main mechanisms leading to insertional mutagenesis for *retroviridae*-based gene delivery models, which comprise retroviral- and lentiviral-derived vector systems [3, 4, 11, 15, 51]. The genera of *retroviridae* have two long terminal repeats (LTRs) situated at the 5′- and 3′-end of their genome (Figure 1). Each LTR contains an enhancer and promoter elements [3]. Consequently, following the integration of the *retroviridae*-based vector into the cellular chromosomes, the 3′-LTR might promote the expression of an endogenous oncogene if this should be situated in the proximity of the integration site [3, 4, 11, 15]. Furthermore, the integration of the *retroviridae*-based vector might silence the expression of cellular tumor suppressor genes if the viral vector integration should occur within an exon of a certain tumor suppressor gene (Figure 2) [3, 4, 11, 15]. This first mechanism that may activate the expression of cellular oncogenes and/or silence tumor suppressor genes has a short range of action. A second mechanism for insertional mutagenesis with a much longer range of action envisions the interaction between the enhancer element of a retroviral LTR and a cellular promoter driving the expression of an endogenous oncogene (Figure 3) [3, 4, 11, 15]. The second mechanism was responsible for the onset of hematological malignancies in five patients of the SCID-X1 gene therapy clinical trials [1, 2, 34–44]. The analysis of malignant cells obtained from the first two leukemic patients of the French SCID-X1 clinical trial showed an overexpression of the LIM only protein 2 (LMO2) oncogene [37, 46]. LMO2 at physiological levels is an important regulator of hematopoietic stem cell development, whereas aberrant levels of LMO2 expression in T cells are associated with acute lymphoblastic leukemia [46, 52–54], whereas the possible involvement of LMO2 overexpression in B-cell acute lymphoblastic leukemia is currently under investigation [55]. A report analyzed the malignant T cells obtained from the first two patients who developed leukemia in the French gene therapy clinical trial for the treatment of SCID-X1 [37]. In one patient, the site of integration of the retroviral vector was 3 kb upstream of the LMO2 transcription start, whereas in the other patient the integration site was in antisense orientation to the LMO2 promoter and 5 kb downstream of the LMO2 transcription site, which corresponded to the first LMO2 intron [37]. This study did not find any replication-competent retrovirus and ruled out a possible contribution of the overexpressed recombinant *γ*c subunit to the onset of the hematological malignancy [37]. According to a couple of reports, an overexpressed recombinant *γ*c subunit induced tumors in animal models [56, 57]. However, further studies on human cells excluded the possibility that the overexpressed recombinant *γ*c subunit had any carcinogenic effects [43, 58–60]. These findings, taken together, indicate that animal models do not always have the ability to recapitulate human maladies, especially in the case of oncological diseases [61–63]. 

LMO2 overexpression, *per se*, might not be sufficient to promote carcinogenesis, which requires a multistep mechanism that involves a variety of genetic alterations, epigenetic mutations, activation of cellular oncogenes, and/or inactivation of putative tumor suppressor genes and environmental factors [61–72]. A subsequent study analyzed the phenotype of malignant cells of the other two leukemic patients who participated in the French SCID-X1 clinical trial [38]. LMO2 overexpression was reported also in these two clinical cases. Moreover, a second retroviral vector integration site was detected in the proximity of the proto-oncogene CCDN2 in leukemic cells of one patient, whereas malignant cells of the other patient carried an integrated retroviral vector close to the proto-oncogene Bmi1 [38]. Blast cells of both leukemic patients exhibited a variety of genetic alterations, such as deletion of tumor suppressor gene cyclin-dependent kinase 2A (CDKN2A), chromosomal translocations, SIL-TAL1 rearrangements, 6q interstitial losses, and gain-of-functions resulting in the activation of NOTCH1 [38]. 

Almost analogous findings were reported in the phenotypic analysis of blast cells of the leukemic patient who was enrolled in the British SCID-X1 gene therapy clinical trial [41]. LMO2 overexpression was observed also in this case, along with a variety of genetic aberrations, which included loss of expression of the tumor suppressor gene CDKN2A, gain-of-function of NOTCH1, and translocation of the T-cell receptor (TCR)-b region to the STIL-TAL1 locus [42]. 

A protocol based on linear amplification-mediated PCR (LAM-PCR) was developed to analyze the retroviral vector integration sites within the human genome following a gene-based intervention in patients [73–76]. The LAM-PCR protocol was used to determine the integration site profile of retroviral-based vectors within the genome of CD34^+^ hematopoietic stem cells of two patients with Wiskott-Aldrich syndrome (WAS), who participated in a phase I gene therapy clinical trial [76–78]. WAS is a genetic disorder characterized by micro-thrombocytopenia, frequent infections, eczema and is associated with a high incidence of lymphoreticular malignancy and autoimmune disorders [79]. The retroviral vector system was utilized to express the functional copy of the human WAS gene in autologous CD34^+^ hematopoietic stem cells, which were transduced *ex vivo *and then injected back into the patients [76, 77]. LAM-PCR analysis was conducted 892 and 891 days after intervention in patients 1 and 2, respectively. This analysis reported 5,709 and 9,538 unique retroviral vector integration sites in patients 1 and 2, respectively. The most recurrent common integration sites (CIS) involved the following genetic *loci*: LMO2, MDS-EVI1, CCDN2 and PRDM16 [76, 77]. Retroviral integration sites either in the proximity or inside the LMO2 and CCDN2 gene *loci* were more frequent in lymphoid cells, whereas retroviral integration sites involving either the PRDM16 and MDS1-EVI1 gene *loci* were predominant in myeloid cells [76, 77]. The reconstitution of the hematopoietic compartment in both patients exhibited polyclonal blood cell populations, which indicates the absence of hematological malignancies [76, 77]. 

Further studies are currently in progress in order to better characterize the mechanism of insertional mutagenesis-induced hematological malignancies in patients. Indeed, the characterization of retroviral vector integration sites in dominating monoclonal blood cell population might reveal important clues on the deregulated cellular signaling systems, which may play a role in the establishment of a malignant cell phenotype and impart clonal expansion [80]. 

## 3. Preclinical Studies for Insertional Mutagenesis

 The use of *retroviridae*-derived vector systems in clinical trials poses a serious safety concern, because of the onset of hematological malignancies that may be promoted by insertional mutagenesis events [1–3]. As already mentioned, insertional mutagenesis-induced malignancies were reported in five leukemic patients of the SCID-X1 gene therapy clinical trials [1–3, 34–44, 51, 81]. Insertional mutagenesis events were also observed both in animal systems [44, 58, 82–84] and human cell culture models [84–91]. These preclinical studies focused on the integrating properties of *retroviridae*-derived vector systems based on murine leukemia virus (MLV), avian sarcoma-leukosis virus (ASLV), and human immunodeficiency virus type 1 (HIV-1) [85–92]. The integration of the aforementioned *retroviridae*-derived vector systems was not completely random within the human genome. In fact, most of integration sites were preferentially localized in the proximity of chromosomal regions that contained transcriptionally active genes [85–91]. In addition, MLV-derived vector systems showed a remarkable predisposition for integration points near transcription start sites [85, 86], whereas HIV-1-based lentiviral vector systems favored genomic *loci* rich in active genes and intercalated with chromosomal regions containing methylated CpG islands that are not permissive for gene expression [85–89]. In contrast, ASLV-based vector systems did not exhibit any specific preference for transcription start sites and had no bias for regions containing active genes [85]. 

 All integrating gene delivery models may present the risk of insertional mutagenesis in target cells. In fact, experiments in animal systems and cell culture models demonstrated that insertional mutagenesis might also be triggered by AAV-based vectors [12] and integrating non-viral vector systems, such as SB DNA transposon-derived vectors [92]. *In vivo *administration of AAV-derived vectors was responsible for either insertional mutagenesis-induced angiosarcomas or hepatocellular carcinomas in mice [12, 92]. Other studies on human cell culture systems detected residual promoter-like properties in terminal repeat sequences of SB DNA transposon-derived vectors, which was able to induce the expression of cellular genes that were contiguous to the integration site of the non-viral vector system [93]. 

 Many studies placed an emphasis on integration of retroviral- and AAV-derived vector systems within common fragile sites of the human genome [90, 94–96]. Human common fragile sites consist of specific regions linked with chromosomal breakpoints, which may play a relevant role in the early stages of malignant cell transformation [95, 97–102]. Interestingly, a number of human oncogenic viruses integrate preferentially their genome within common fragile sites [95]. These human oncogenic viruses comprise human papilloma virus [103–106], Epstein-Barr virus [95, 107, 108], and hepatitis B virus [109]. 

 The analysis of so-called hotspots for certain viral-derived vector systems integration sites is essential to determine the mechanism of insertional mutagenesis-related onset of malignancies in patients. 

## 4. Tactics to Reduce the Incidence of Insertional Mutagenesis

 Gene therapists are currently addressing the issue of insertional mutagenesis-induced malignancies in patients by pursuing three main approaches: (i) engineering of safer integrating gene delivery models; (ii) development of episomal gene delivery systems with improved duration of transgene expression in transduced cell populations; (iii) production of genetically modified meganucleases and zinc-finger proteins for the correction of genetic defects. 

**Figure 4:**
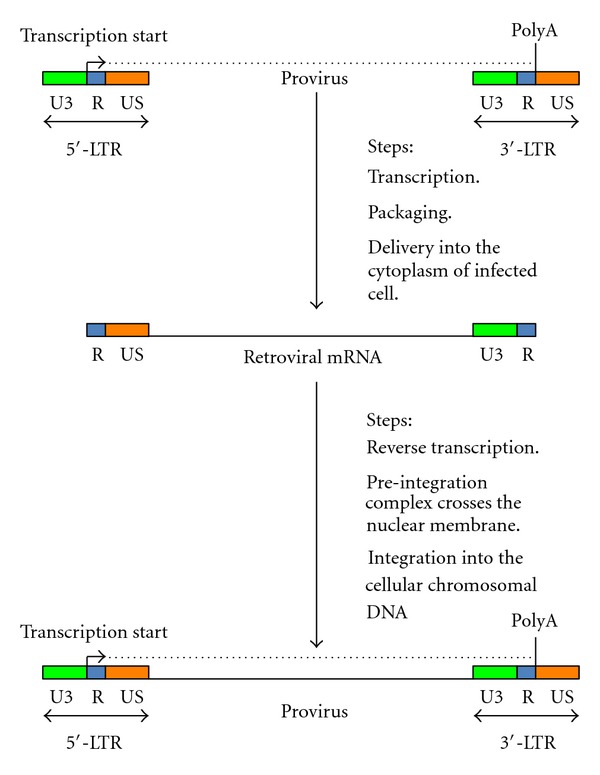
This figure illustrates the replication cycle of the genera of *retroviridae.* As shown at the top of the figure, the proviral DNA transcribes the retroviral mRNA, which is then packaged into the virion. The retroviral mRNA contains only one copy of the U3 and U5 regions. After the virus enters the target cells, the retroviral mRNA is released in the cellular cytoplasm and reverse transcribed. During this process, the U3 region present at the 3′-end of the retroviral mRNA is duplicated at the 5′-end of the newly synthesized proviral DNA, whereas the U5 region in the 5′-end of the retroviral mRNA is copied at the 3′-end of the proviral DNA. The proviral DNA associates with other retroviral and cellular factors to form the preintegration complex, which can cross the nuclear membrane and integrate into the infected cell's genome.

 The central issue for the development of safer integrating gene delivery systems consists of producing gene transfer shuttle vectors that do not affect the physiological genomic organization of transduced cells. Therefore, it is necessary to prevent interactions between enhancer and/or promoters of the vector system and the host genome. The engineering of self-inactivating *retroviridae*-derived vectors may minimize the incidence of insertional mutagenesis-related events in transduced cells [3, 11, 15]. The replication cycle of the genera of *retroviridae* comprises an initial transcription stage, in which the viral messenger RNA (mRNA) is synthetized and subsequently packaged into the virion (Figure 4). This viral mRNA does not contain the enhancer/promoter U3 region in the 5′-LTR and the U5 region in the 3′-LTR. After cell infection, the viral mRNA is released into the cytoplasm and then is reversed transcribed into double stranded proviral DNA. At this stage, the U3 region in the 3′-LTR is copied at the 5′-LTR, whereas the U5 region in the 5′-LTR is copied at the 3′-LTR. The double-stranded proviral DNA is successively assembled in a preintegration complex, which crosses the nuclear membrane and is integrated within the genome of the infected cell (Figure 4) [3, 11, 15]. 

**Figure 5:**
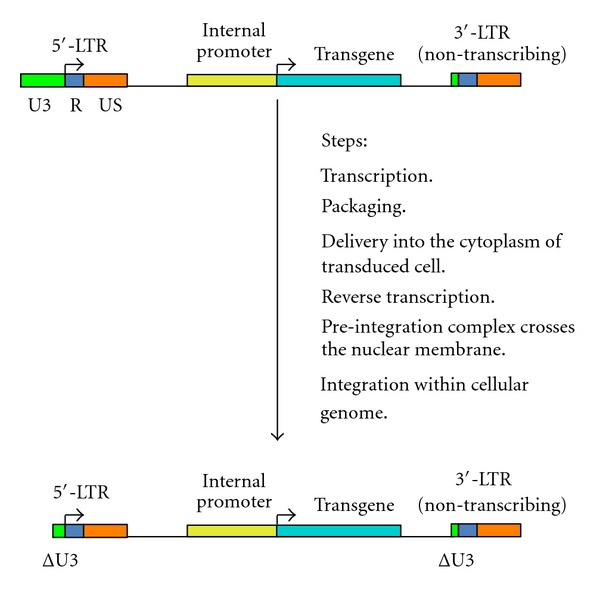
Self-inactivating *retroviridae*-based vector systems can be engineered via deletion of the viral enhancer/promoter U3 region in the 3′-LTR (upper part of the figure). This deletion ultimately results in a proviral form with two nontranscribing LTRs in transduced cells (lower part of the figure). At this point, only the internal promoter is able to transcribe the mRNA encoding for the transgene.

 Self-inactivating *retroviridae*-derived vectors are produced by a deletion of the U3 region in the 3′-LTR (Figure 5) [3, 11, 15]. Thus, the 3′-LTR is no longer transcribing. In this context, the 5′-LTR drives the transcription of a viral mRNA lacking the U3 region in the 3′-LTR. Following the reverse transcription process in transduced cells, the truncated U3 region in the 3′-LTR is reproduced at the 5′-LTR. This results in a proviral DNA without transcriptional activity at both LTRs (Figure 5). Obviously, self-inactivating *retroviridae*-derived vectors need an internal promoter to express the transgene (Figure 5) [3, 11, 15]. 

 Self-inactivating *retroviridae*-derived vectors may be considered safer than their original counterparts, which are termed gamma-retroviral or gamma-lentiviral based vectors [3, 11, 15]. The deletion of retroviral enhancers and promoter regions in the LTRs minimizes the probability of interactions between the transfer vector system and the host genome [3, 11, 15]. In fact, the nontranscribing 3′-LTR is not able to drive the expression of cellular oncogenes that might be present in the vicinity of the integration site (Figure 5). Moreover, the removal of *retroviridae* enhancer regions precludes the long-range interference with cellular promoters that control the expression of endogenous oncogenes (Figure 5) [3, 11, 15]. However, the presence of an enhancer within the internal promoter of a self-inactivating *retroviridae*-derived vector still poses the issue of insertional mutagenesis, as it may stimulate cellular promoters of oncogenic factors [3, 11, 15, 110, 111]. Studies produced self-inactivating lentiviral vectors containing enhancer-less internal promoters [110, 111]. In one case, a self-inactivating lentiviral vector system utilized as internal promoter the enhancer-less and methylation-free CpG islands promoter of the ubiquitously acting chromatin opening elements, which is a human housekeeping gene and was termed A2UCOE [110, 112]. The use of the A2UCOE internal promoter has a dual advantage: the first advantage is related to the vector design safety improvement that is due to the absence of enhancer regions; the second advantage is correlated with the absence of CpG-rich islands within the sequence of the human housekeeping gene promoter, which confers resistance to *de novo* methylation-mediated transcriptional silencing [110, 112]. The self-inactivating *retroviridae*-derived vector containing the enhancer-less A2UCOE internal promoter was utilized for the gene transduction of the recombinant *γ*c subunit in primary bone-marrow-derived CD34^+^ hematopoietic stem cells of a patient with SCID-X1 [110]. Transduced cell populations readily expressed the recombinant *γ*c subunit and became susceptible to the action of interleukins in the *in vitro* system [110]. Similar results were observed in a SCID-X1 mouse model [110]. These findings indicate that the production of enhancer-less self-inactivating *retroviridae*-derived vectors constitute a significant improvement in safety design. 

 In other studies, the internal promoters of self-inactivating lentiviral vector systems were based either on the enhancer-less human Vav1 promoter [111, 113] or the phosphoglycerate kinase (PGK) promoter [113]. The enhancer-less human Vav1 promoter was used for the expression of the recombinant *γ*c subunit in a murine model of SCID-X1 [111]. Although the transgene expression levels were not high, it was possible to correct the phenotype of the disease in a mouse model [111]. 

 Another preclinical study on Fanconi anemia utilized the enhancer-less human Vav1 promoter and the phosphoglycerate kinase (PGK) promoter [113]. Fanconi anemia is an inherited genetic disorder leading to bone marrow failure and high incidence of leukemia [114]. Genotypic analysis of patients with Fanconi anemia identified mutations in a group of fifteen genes, which were termed FANC genes [114]. Mutations in the so-called FANCA gene are among the most frequent in patients with Fanconi anemia [114]. Self-inactivating lentiviral vector system was utilized in a preclinical study to express the functional copy of FANCA, which was driven either by the enhancer-less human Vav1 promoter or the phosphoglycerate kinase (PGK) promoter [113]. Transduction efficiency and duration of transgene expression were efficient in this study. However, the levels of transgene expression were rather low. In order to optimize FANCA expression levels, the investigators utilized a variant of the woodchuck hepatitis virus posttranscriptional regulatory element (WPRE) region [113]. Overall, all tested vector systems induced a comparable phenotypic correction in cell culture models for Fanconi anemia [113]. 

 A self-inactivating lentiviral vector was utilized in a mouse model for the correction of SCID-X1. This vector system used a minimal promoter obtained from the eukaryotic elongation factor *α* (EF1*α*) gene to drive the expression of a codon-optimized human *γ*c cDNA [115]. Remarkably, this vector system was able to correct the phenotype of the disease in the mouse model, without causing the overexpression of the LMO2 oncogene [115]. In contrast, the self-inactivating lentiviral vector system containing the human *γ*c promoter driving the expression of the human *γ*c cDNA induced the over-expression of LMO2 [115]. The chicken hypersensitivity site 4 (cHS4) insulator was placed in the 3′-LTR of both vector systems [115]. Insulators were utilized to prevent the silencing of *retroviridae*-encoded transgenes, which may derive either from *de novo* methylation of viral promoters and/or position effects imparted by chromosomal sequences surrounding the retroviral integration site [116, 117]. However, the result of this study indicates that insulators are not efficacious in providing a barrier against the genotoxicity of *retroviridae*-based vectors [115]. Studies are currently in progress in order to identify novel insulators that have the ability to prevent genotoxic effects of integrating gene delivery systems [118]. 

**Figure 6:**
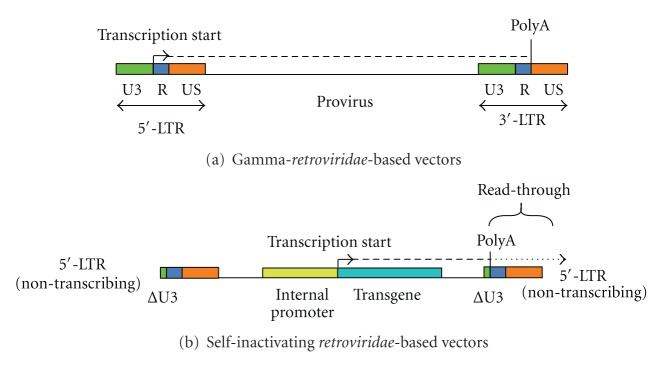
This figure shows a comparison between gamma-*retroviridae*- and self-inactivating-*retroviridae*-derived vector systems. The polyA signal is a more efficient transcription termination signal in the gamma-*retroviridae*-derived vector than in the self-inactivating variant.

 A major inadequacy of self-inactivating *retroviridae*-derived vectors consists of an increased polyadenylation (polyA) signal read-through in the 3′-LTR (Figure 6) [119]. Leaky transcriptional termination signals may result in the activation of silent cellular oncogenes in transduced cells (Figure 6) [119]. Furthermore, the polyA read-through in packaging cell lines might cause the uptake of cellular oncogenes into the genome of *retroviridae*-based vectors, which, in turn, may transmit it to transduced cell populations [119]. This phenomenon occurred in so-called acute transforming retroviruses [119–122]. The deletion of the U3 region from the 3′-LTR enhances the probability of polyA read-through in self-inactivating *retroviridae*-based vectors [123, 124]. In fact, the U3 region of the LTR contains both transcription termination motifs and enhancer-promoter elements [123, 124]. In this respect, a study demonstrated that the incorporation of seven SV40-derived upstream polyA enhancer elements in the residual U3 region improved transcription termination efficiency in self-inactivating *retroviridae*-based vectors [119]. This study reported also a 3-fold enhancement in viral vector titers, along with increased and stable transgene expression levels in transduced cell populations [119]. 

 Insertional mutagenesis-induced malignancies were also observed in animal models for AAV-mediated gene transfer [12]. Wild-type AAV integrates specifically into a safe site of the human genome [12]. This safe integration site is termed AAV1 and is located in the q arm of the chromosome 19, between q13-3 and qter [3, 12]. The wild-type AAV-specific integration into AAV1 is mediated by the viral factors Rep 68 and Rep 78 [3, 12]. However, most of the wild-type AAV genome must be removed in order to engineer AAV-derived vector systems, because of the limited capacity of AAV-based vectors in accommodating transgenes [3, 4, 12]. For this reason, AAV-derived vectors do not have Rep 68 and Rep 78 proteins and, therefore, integrate randomly within the cellular genome [3, 4, 12]. The field of vector design is attempting to restore AAV1-specific integration via coexpression in transduced cell populations of AAV-based vectors along with Rep 68 and Rep 78 proteins [12]. 

 Another strategy to circumvent the issue of insertional mutagenesis consists of producing episomal gene delivery models with enhanced duration of transgene expression. On one hand, episomal vector systems do not alter the transduced cell genome, as they do not integrate into the chromosomal DNA [14]. On the other hand, nonintegrating gene delivery models only allow for a transient expression of the transgene [14]. On these grounds, the field of vector design is working on the development of novel systems for the stabilization of transgene expression after the episomal vector-mediated gene transfer into target cells. To this end, gene therapists placed a particular emphasis on the development on episomal lentiviral vectors [125–135], because of their ability to transduce proficiently an ample variety of mammalian cells and regardless of their cell cycle phase [11]. Several studies showed that episomal lentiviral-based vectors maintained high transduction efficacy and wide cell tropism [125, 128–131]. Studies on mouse models focused on *in vivo* gene delivery of episomal lentiviral vectors in the central nervous system [128], stem cells of the hematopoietic compartment [130], muscles [131], and ocular tissues [135]. Episomal lentiviral vector systems can be produced via inactivation of the viral integrase and mutation of the integrase attachment sites (att) that are present in the U3 region of the 5′- and 3′-LTRs [125]. The viral integrase is inactivated by site directed mutagenesis of the chromosome-binding moiety, proviral DNA binding domain and catalytic site [125, 130]. These point mutations do not affect at all the ability of the viral integrase to transport the pre-integration complex through the nuclear membrane [125, 130]. Negligible levels of residual integration of episomal lentiviral vectors were reported in some transduced cell populations [124]. Nevertheless, such a residual integration activity was related to background recombination events, rather than the mutated viral integrase [125]. Episomal lentiviral vector systems allow for a better long-term transgene expression in nondividing cells than in dividing cells, as mitosis may cause the dilution of the episomal vector genome in progeny cells [125, 128, 131]. Remarkably, the inclusion of scaffold/matrix attachment regions (S/MAR) may increase the duration of transgene expression of episomal lentiviral vector systems within transduced cell populations [136, 137]. This approach may also be utilized to enhance the episomal permanence of non-viral gene-delivery models in transfected human cells [137, 138]. In fact, promising results were reported in human hematopoietic progenitor cells [138]. However, the efficacy of S/MAR in increasing episomal permanence was cell-type-dependent [138]. Further research is necessary to improve the preclinical applications of nonintegrating gene delivery models. 

 Meganucleases and artificially engineered zinc-finger proteins are attracting a great deal of interest in the field of gene transfer technology, as they hold the potential of conducting either site-specific rectification of defective genes, or inclusion of genetic elements into selected *loci* within the genome of transfected cells [139–143]. ZFPs have the ability to identify several varieties of DNA motifs [139–142]. The C_2_H_2_-type ZFPs are by far the most common DNA-binding moieties of transcription factors [139]. Amazingly, such DNA-binding domains constitute about 2% of the entire human genome [139–144]. Novel C_2_H_2_-type ZFPs can be designed for the binding of specific DNA sequences. This strategy relies on the variation of C_2_H_2_-type ZFPs that are present in the Sp1 transcription factor [139]. A similar tactic was utilized for the production of recombinant meganucleases or homing endonucleases [143, 145–148]. 

 Meganucleases consist of sequence-specific endonucleases that identify relatively large DNA binding motifs in living cells [145]. Such DNA binding motifs are in the range of 14 base pairs (bp) or more [145]. The production of genetically modified meganucleases might allow for the design of site-specific gene delivery into the human genome [145–148]. For example, the I-CreI-derived meganuclease DNA binding domain was adjusted to recognize specifically new designed genetic sequences [145–147]. The artificial I-CreI-derived meganuclease was utilized to target the human xeroderma pigmentosum group C (XPC) gene, whose genetic mutations may be responsible for the pathogenesis of Xeroderma Pigmentosa disease [146], which is a rare autosomal recessive genetic illness causing both hypersensitivity to ultraviolet light and high incidence of skin cancer [149]. Two independent reports showed an efficient I-CreI-derived meganuclease-mediated rectification of a flawed chromosomal locus in various mammalian cell lines [146, 148]. Interestingly, the employment of genetically engineered meganucleases was not associated with noticeable levels of genotoxicity in mammalian cell lines [143, 148]. Other studies are currently underway for the characterization of I-SceI [150–152] and I-DmoI meganucleases [152]. 

## 5. Conclusion

The possible onset of insertional mutagenesis-induced malignancies in patients constitutes a serious obstruction to the establishment of gene therapy programs in the clinical setting. Undeniably, gene transfer technology holds enormous therapeutic potential for the treatment of a wide variety of pathological conditions, such as cancer, cardiovascular disorders, genetic diseases, diabetes, neurodegenerative illnesses, and infectious maladies [3, 4]. This is the reason that motivates a keen interest for gene therapy. Moreover, the merging between gene transfer technology and stem cell research may have important implications in the area of regenerative medicine.

The field of vector design is currently tackling the critical issue of insertional mutagenesis by adopting three main strategies. The first approach is based on the production of integrating gene delivery models that can only express the transgene and do not interact with the human genome, in order to avoid the activation of cellular oncogenes. In this respect, enhancer-less self-inactivating *retroviridae*-derived vectors have attracted a lot of interest and seem very promising. The second approach depends on the engineering of episomal vector systems, which were genetically modified to allow for a more stable and long-lasting transgene expression in transduced cell populations. The third approach utilizes genetically modified meganucleases and artificially engineered zinc-finger proteins to carry out either site-specific correction of faulty genes or inclusion of genetic factors into specific and safe *loci* of the genome of transfected cells. These strategies require major efforts from gene therapists. However, the solution of the insertional mutagenesis issue may bring a substantial contribution to a more successful application of gene therapy programs in various sectors of experimental medicine.
